# Ultrasound-Guided Transversalis Fascia Plane Block Versus Intrathecal Morphine for Post-Cesarean Analgesia: A Prospective Observational Comparative Cohort Study Incorporating a Non-Inferiority Analytical Framework

**DOI:** 10.3390/jcm15114157

**Published:** 2026-05-28

**Authors:** Ilke Tamdogan, Ibrahim Yilmaz

**Affiliations:** 1 Department of Anesthesia and Reanimation, Giresun University Faculty of Medicine, Giresun 28200, Turkey; ilke.tamdogan@giresun.edu.tr; 2 Unit of Pharmacovigilance, Dr. Ismail Fehmi Cumalioglu City Hospital, Ministry of Health of the Republic of Turkey, Tekirdag 59020, Turkey

**Keywords:** caesarean delivery, post-operative analgesia, transversalis fascia plane block, intrathecal morphine, regional anesthesia, ObsQoR-11, opioid-sparing

## Abstract

**Background:** Intrathecal morphine (ITM) is a widely used reference approach for post-caesarean analgesia but is associated with neuraxial opioid-related side effects, particularly pruritus and nausea. The transversalis fascia plane (TFP) block is a relatively recent regional technique targeting the transversalis fascia; however, comparative effectiveness data in the obstetric setting remain limited. This study aimed to evaluate whether TFP block provides analgesic outcomes broadly similar to those observed with ITM with respect to 24 h rescue analgesic requirement following elective caesarean delivery under spinal anesthesia, and to compare patient-centered recovery quality. **Methods:** This prospective, single-center, observational comparative cohort study was conducted in a tertiary obstetric unit. Sixty American Society of Anesthesiologists (ASA) physical status II parturients undergoing elective caesarean delivery under spinal anesthesia were included. Postoperative analgesic strategy was determined according to routine clinical practice, with patients receiving either ITM (100 µg) or bilateral ultrasound-guided TFP block with 0.25% bupivacaine. The primary outcome was cumulative tramadol consumption within the first 24 h postoperatively. Secondary outcomes included Numerical Rating Scale (NRS) pain scores at rest and on movement at 0, 3, 6, 12, and 24 h; postoperative nausea and vomiting (PONV) and pruritus scores; and Obstetric Quality of Recovery-11 (ObsQoR-11) scores at 24 and 48 h. A non-inferiority analytical framework was applied to the primary outcome. **Results:** Both groups had a median tramadol consumption of 0 mg (interquartile range (IQR) 0–0). Rescue analgesic rates were 23.3% (ITM) versus 16.7% (TFP; OR 0.66, 95% CI 0.18–2.36; *p* = 0.748). Within an exploratory observational non-inferiority analytical framework, the findings were consistent with non-inferiority of TFP block; however, these analyses should be interpreted as hypothesis-generating rather than confirmatory (risk difference −6.7 percentage points; upper 95% CI +13.5% relative to a prespecified margin of +15%). TFP block was associated with higher ObsQoR-11 scores at 24 h (median 96 vs. 88; *p* = 0.010; Cliff’s δ = −0.39) and 48 h (median 96 vs. 91; *p* = 0.017; Cliff’s δ = −0.36). Pruritus at 6 and 12 h was nominally lower in the TFP group. **Conclusions:** In this prospective observational cohort, TFP block was associated with analgesic outcomes broadly similar to those observed with ITM, with exploratory differences in patient-centered recovery measures. Within the applied exploratory observational analytical framework, these findings were broadly consistent with non-inferiority; however, given the observational design, results should be interpreted cautiously, and the present study does not establish formal non-inferiority or equivalence. TFP block may represent a potential opioid-sparing option warranting confirmation in adequately powered randomized studies.

## 1. Introduction

Caesarean delivery is among the most commonly performed surgical procedures worldwide, and inadequate postoperative analgesia remains an important contributor to maternal morbidity, impaired early neonatal bonding, and delayed functional recovery [1]. Optimal post-caesarean analgesia therefore requires not only effective nociceptive control but also minimization of opioid-related adverse effects, which may interfere with maternal recovery and early neonatal care. In this context, contemporary perioperative care increasingly emphasizes patient-centered recovery as a multidimensional construct, incorporating functional capacity, emotional well-being, and the ability to participate in early neonatal care, rather than pain intensity alone.

Intrathecal morphine (ITM), administered at the time of spinal anesthesia, remains the reference standard for post-caesarean analgesia due to its well-established efficacy in reducing postoperative opioid requirements and providing prolonged analgesia for up to 24 h [2,3]. Its use is supported by major obstetric anesthesia guidelines [4,5]. However, neuraxial opioid administration is consistently associated with dose-dependent adverse effects, including pruritus, nausea and vomiting, and, less commonly, respiratory depression [6,7]. These adverse effects are thought to be related to central opioid receptor activation following neuraxial opioid administration rather than peripheral nociceptive modulation, and may adversely affect overall recovery despite adequate analgesia. Increasing attention has therefore been directed towards validated recovery instruments such as the Obstetric Quality of Recovery-11 (ObsQoR-11), which capture clinically relevant dimensions of postoperative recovery beyond pain scores alone [8].

In response to these limitations, regional anesthetic techniques have been increasingly incorporated into multimodal analgesic strategies. The transversus abdominis plane (TAP) block has been widely evaluated for post-caesarean analgesia, although its incremental benefit in the presence of neuraxial opioids appears variable and context-dependent [9]. More recently, the transversus fascia plane (TFP) block has been described as a subfascial technique targeting the plane between the transversus abdominis muscle and the transversalis fascia, providing somatic analgesia to the anterior abdominal wall, including the Pfannenstiel incision site [10,11]. By depositing local anesthetic in proximity to nerves traversing the transversalis fascia, the TFP block has been proposed as a fascial plane technique with potential cephalad spread beyond the conventional TAP [12].

Other regional techniques, including quadratus lumborum (QL) block, have also been extensively investigated; however, reported benefits over neuraxial opioid-based strategies remain inconsistent across studies, particularly when multimodal systemic analgesia is applied [13,14]. Recent high-quality evidence suggests that fascial plane blocks may provide analgesic efficacy approaching that reported with ITM in selected settings, although findings remain heterogeneous and context-dependent [15].

The clinical question in contemporary obstetric anesthesia is therefore no longer limited to whether regional techniques reduce postoperative opioid requirements, but rather whether they meaningfully influence patient-centered recovery. Adequate analgesia does not necessarily translate into optimal recovery, particularly when opioid-related adverse effects interfere with mobilization, maternal comfort, and interaction with the newborn [6,7,8]. Conversely, while fascial plane blocks may reduce opioid exposure, the extent to which they confer clinically meaningful advantages in recovery outcomes remains uncertain, with existing evidence characterized by methodological heterogeneity and variability in outcome selection [13,14,15].

An additional consideration is the distinction between efficacy demonstrated in controlled trial environments and effectiveness observed in routine clinical practice. Randomized controlled trials provide high internal validity but are typically conducted under controlled conditions with strict inclusion criteria, which may limit generalizability. In contrast, observational analyses of real-world practice may better reflect the complexity of perioperative care, including variability in patient characteristics, clinician decision-making, and institutional protocols. Such analyses, however, are inherently susceptible to confounding and bias, and their findings should therefore be interpreted cautiously and regarded as hypothesis-generating.

Within this framework, the comparative effectiveness of TFP block and ITM in routine obstetric practice remains insufficiently characterized. Although both approaches are integrated into multimodal analgesic pathways, direct comparative data—particularly those incorporating patient-centered recovery outcomes—remain limited. Notably, recent randomized evidence evaluating comparative fascial plane approaches has begun to explore recovery-focused endpoints, although findings remain preliminary and require contextual interpretation [16].

The present prospective observational comparative cohort study was therefore undertaken to evaluate whether bilateral ultrasound-guided TFP block is associated with analgesic outcomes broadly similar to those observed with ITM following elective caesarean delivery, and to assess whether any between-group differences extend to patient-centered recovery outcomes. In addition, a non-inferiority analytical framework was applied to the primary outcome to support comparative interpretation.

## 2. Materials and Methods

### 2.1. Study Design and Setting

This prospective, single-center, observational comparative cohort study was conducted at a tertiary obstetric unit. The study was designed to evaluate postoperative analgesic effectiveness and patient-centered recovery outcomes in parturients undergoing elective caesarean delivery under spinal anesthesia under routine clinical practice conditions. Participants were followed prospectively, and outcomes were compared between exposure groups defined according to the postoperative analgesic strategy applied in routine care. The study was conducted and reported in accordance with the Strengthening the Reporting of Observational Studies in Epidemiology (STROBE) guidelines [17].

### 2.2. Ethical Approval

The study protocol was approved by the Clinical Research Ethics Committee of Ondokuz Mayis University Faculty of Medicine (Decision No. 2025/581). The study was registered at ClinicalTrials.gov (NCT07374133). All procedures were conducted in accordance with the principles of the Declaration of Helsinki. Written informed consent was obtained from all participants prior to study inclusion. Although the study was prospectively conducted with prior ethics committee approval, ClinicalTrials.gov registration was completed on 18 March 2026, after enrollment had already begun, because the observational design was initially considered part of routine clinical practice assessment. The study was subsequently registered retrospectively to improve transparency and reporting compliance.

### 2.3. Patient Selection

Eligible patients were consecutively screened from the population of parturients undergoing elective caesarean delivery at Giresun Maternity and Children’s Hospital between February and March 2026. Adult patients (≥18 years) with American Society of Anesthesiologists (ASA) physical status II, singleton pregnancy, and scheduled elective caesarean delivery under spinal anesthesia were considered eligible for inclusion. Patients were excluded in the presence of emergency caesarean delivery, conversion to general anesthesia, chronic opioid use or pre-existing pain disorders, contraindications to neuraxial anesthesia or regional block, known allergy to study medications, or incomplete outcome data. Participants were categorized into exposure groups according to the postoperative analgesic strategy applied in routine clinical practice, including ITM or bilateral ultrasound-guided TFP block.

### 2.4. Group Definition and Exposure Classification

Given the observational design of the study, no randomization or allocation procedures were performed. Participants were assigned to postoperative analgesic strategies as part of routine clinical care, based on the responsible anesthesiologist’s clinical judgment and patient-related factors. Accordingly, participants were categorized into two exposure groups: those receiving ITM as part of spinal anesthesia and those receiving bilateral ultrasound-guided TFP block in the postoperative period. Outcome assessment was performed by an anesthesia resident not involved in the anesthetic management or block procedures. Due to the nature of the interventions, blinding of the treating anesthesiologist was not feasible; however, efforts were made to ensure that outcome assessment was conducted without knowledge of group allocation where possible.

### 2.5. Anesthetic and Analgesic Management

All patients underwent standard spinal anesthesia according to institutional practice. Spinal anesthesia was performed in the sitting position using 12 mg of hyperbaric bupivacaine (Marcaine Spinal Heavy 0.5%, AstraZeneca, Cambridge, UK) combined with 20 µg fentanyl (Talinat^®^, Vem Ilac, Ankara, Turkey). Intrathecal fentanyl was administered uniformly in both groups as part of the institution’s standard spinal anesthesia regimen to optimize intraoperative anesthesia quality. In patients receiving ITM, an additional 100 µg of preservative-free morphine (Galen Ilac, Istanbul, Turkey) was administered as part of the intrathecal injectate. The selected intrathecal morphine dose and bupivacaine regimen reflected routine institutional obstetric anesthesia practice during the study period. In patients receiving TFP block, a bilateral ultrasound-guided TFP block was performed at the end of surgery with the patient in the supine position. Using a high-frequency linear transducer with an ultrasound system (HM70 EVO, Samsung Medison Co., Ltd., Seoul, Republic of Korea), the plane between the transversus abdominis muscle and the transversalis fascia was identified, and the block was performed using a 20G × 100 mm echogenic block needle (Stimuplex^®^ Ultra 360^®^, B. Braun, Melsungen, Germany) with an in-plane technique. Following hydrodissection with saline to confirm correct needle placement, 20 mL of 0.25% bupivacaine was injected on each side. Postoperative analgesia in both groups was based on a standard multimodal regimen, including scheduled paracetamol and non-steroidal anti-inflammatory drugs. Rescue analgesia with intravenous tramadol was administered as required based on patient-reported pain intensity and clinical judgment, typically when NRS scores were ≥4.

### 2.6. Outcomes

The primary outcome of the study was the proportion of patients requiring rescue analgesia within the first 24 postoperative hours. Cumulative tramadol consumption (mg) was analyzed as a supportive secondary outcome. Secondary outcomes included postoperative pain intensity assessed using the NRS (0 = no pain, 10 = worst imaginable pain) at rest and during movement at predefined time points (0, 3, 6, 12, and 24 h after surgery). In addition, the incidence and severity of postoperative nausea and vomiting (PONV) and pruritus were recorded at the same time intervals using standard ordinal scales. Patient-centered recovery was evaluated using the validated ObsQoR-11 questionnaire, with scores ranging from 0 to 110, where higher scores indicate better recovery. ObsQoR-11 assessments were performed at 24 and 48 h postoperatively. All outcome variables were prospectively defined prior to data collection and were assessed using standardized protocols. The selection of outcomes was guided by clinically relevant domains of postoperative recovery, including analgesia, opioid-related adverse effects, and functional recovery.

### 2.7. Statistical Analysis

Statistical analyses were performed using SPSS version 30 (IBM Corp., Armonk, NY, USA). Continuous variables were assessed for normality using the Shapiro–Wilk test and visual inspection of histograms. Data are presented as mean ± standard deviation (SD) or median [interquartile range (IQR)], as appropriate. Categorical variables are presented as counts and percentages.

Between-group comparisons were performed using the Mann–Whitney U test for continuous or ordinal variables and the chi-square or Fisher’s exact test for categorical variables, as appropriate. Given the expected zero-inflated distribution of postoperative tramadol consumption, the primary outcome was evaluated using a two-part analytical approach: (i) comparison of the proportion of patients requiring rescue analgesia, and (ii) comparison of cumulative tramadol dose among those requiring rescue analgesia.

Secondary outcomes, including repeated measurements of pain scores, PONV, and pruritus, were initially analyzed at individual time points. In addition, exploratory repeated-measures analyses were performed to assess overall group effects and the consistency of between-group differences across time. To aid clinical interpretation, effect sizes were reported where appropriate. For ObsQoR-11, the Hodges–Lehmann estimator was used to estimate the median difference between groups.

Additional exploratory analyses were conducted to further characterize between-group differences, including a non-inferiority assessment for rescue analgesic requirement, adjusted logistic regression analysis, and estimation of clinically meaningful differences based on established thresholds. To account for multiple comparisons across secondary outcomes, Holm–Bonferroni correction was applied.

Primary analyses were conducted using SPSS, while additional advanced statistical analyses were performed using Python version 3.13 (Python Software Foundation, Wilmington, DE, USA) with the statsmodels, SciPy, and pingouin libraries.

A two-sided *p*-value < 0.05 was considered statistically significant for primary analyses; secondary outcomes were interpreted in the context of multiplicity adjustment. Results of exploratory analyses were interpreted cautiously and considered supportive rather than confirmatory.

In addition, a priori sample size considerations were evaluated for the primary non-inferiority endpoint. A priori sample size calculation was performed for the primary non-inferiority endpoint (rescue analgesic requirement within 24 h). Assuming an expected event rate of approximately 20–25% in the ITM group, a non-inferiority margin of 15 percentage points, a one-sided α of 0.025, and a power of 80%, the required sample size was estimated to be larger than the feasible sample available during the study period. Therefore, a pragmatic sample of 30 patients per group was enrolled, and the study should be interpreted as exploratory, particularly for secondary outcomes.

For consistency, continuous outcomes are reported as rounded median values in the main manuscript, while exact values are retained in the Supplementary Materials.

### 2.8. Use of Generative AI and AI-Assisted Technologies

Minor language editing and formatting assistance were obtained using AI-based tools. All scientific content, statistical analyses, interpretations, and manuscript revisions were independently developed, verified, and approved by the authors.

## 3. Results

### 3.1. Patient Characteristics

Sixty patients were enrolled and included in the analysis (30 per group). All patients completed the 24 h follow-up; 48 h ObsQoR-11 data were available for all 60 participants. Baseline demographic and clinical characteristics were comparable between groups (Table 1).

Mean age was 29.6 ± 5.3 years in the ITM group and 29.8 ± 5.0 years in the TFP block group (*p* = 0.970). BMI, weight, height, and number of prior caesarean deliveries were comparable between groups (all *p* > 0.2). Prior caesarean deliveries were 1.9 ± 0.7 in the ITM group and 1.8 ± 0.6 in the TFP block group (*p* = 0.568).

### 3.2. Primary Outcome: Rescue Analgesic Requirement and Consumption

The primary outcome was rescue analgesic requirement within 24 h postoperatively (Table 2; *p* = 0.748). Total 24 h tramadol consumption in the overall cohort was analyzed as a supportive secondary measure.

**Figure 1:**
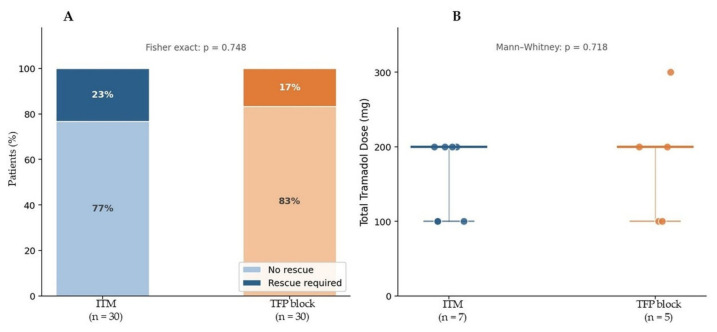
Rescue Analgesic Consumption. (**A**): Proportion of patients requiring rescue tramadol within 24 h. Stacked bar charts indicate percentages with (dark fill) and without (light fill) rescue analgesia; Fisher’s exact test was used for comparison. (**B**): Total cumulative tramadol dose (mg) among patients requiring rescue analgesia only (ITM *n* = 7; TFP *n* = 5); individual data points are shown with median and IQR markers; Mann–Whitney U test was used.

Rescue analgesia was required by 7 of 30 patients (23.3%) in the ITM group and 5 of 30 (16.7%) in the TFP block group (OR 0.66, 95% CI 0.18–2.36). Among patients requiring rescue analgesia, cumulative tramadol doses were similar between groups, with a median of 200 mg in both groups (IQR 100–200 mg; *p* = 0.718; Figure 1).

Non-inferiority analysis yielded a risk difference of −6.7 percentage points (95% CI −26.8% to +13.5%). The upper bound of the 95% confidence interval (+13.5%) remained below the prespecified non-inferiority margin of +15 percentage points (one-sided *p* = 0.018). These findings were consistent with non-inferiority of TFP block within the applied analytical framework.

Adjusted logistic regression analysis including maternal age, BMI, and parity showed no evidence of meaningful confounding (adjusted OR 0.72, 95% CI 0.19–2.68; *p* = 0.623).

A summary of effect sizes across outcomes is shown in Figure S1. Detailed regression analyses are provided in the Supplementary Materials (Table S1 and Figure S2). Non-inferiority analytical framework results are presented in Table S2, with graphical illustration in Figure S3.

### 3.3. Secondary Outcomes: NRS Pain Scores

**Figure 2:**
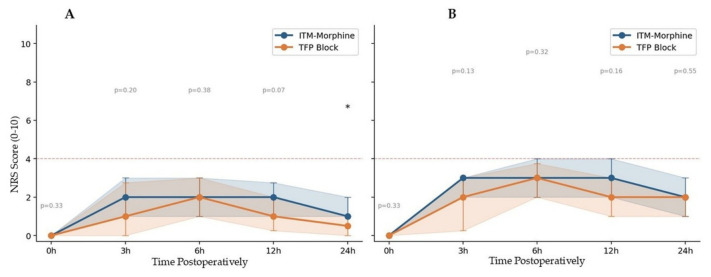
NRS Pain Scores Over Time. Median NRS pain scores (0–10) with interquartile ranges (shaded areas) at rest (**A**) and on movement (**B**) in the ITM (*n* = 30) and TFP block (*n* = 30) groups at 0, 3, 6, 12, and 24 h postoperatively. The dashed red line indicates the rescue analgesic threshold (NRS ≥ 4). Statistical comparisons were performed using the Mann–Whitney U test (two-sided), with raw *p*-values presented. * *p* < 0.05 (nominal significance prior to multiplicity correction).

NRS pain scores at rest and on movement were uniformly low in both groups throughout the observation period and remained below the rescue threshold of 4 at all assessed time points (Table 3; Figure 2).

At 24 h, resting NRS scores were nominally lower in the TFP block group (median 0 [IQR 0–1]) compared with the ITM group (median 1 [IQR 1–2]; *p* = 0.022); however, this difference did not remain significant after Holm–Bonferroni correction for multiple comparisons (corrected *p* = 0.423).

Exploratory repeated-measures analysis across all time points suggested an overall group effect for NRS at rest (Stouffer z = 2.79, *p* = 0.005) and NRS on movement (z = 2.09, *p* = 0.036), with no evidence of a group × time interaction (*p* = 0.684 and 0.883, respectively), suggesting that observed between-group differences were consistent across the postoperative period rather than driven by a single time point.

Comprehensive repeated-measures results and multiplicity-adjusted analyses are presented in the Supplementary Materials (Figure S4; Tables S3 and S4).

### 3.4. Secondary Outcomes: PONV and Pruritus

**Figure 3:**
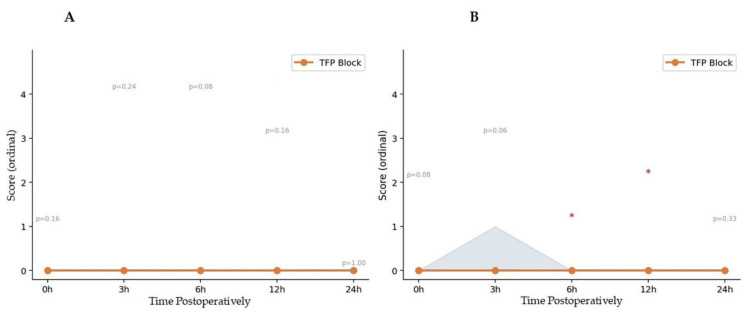
PONV and Pruritus Scores Over Time. Median ordinal scores for PONV ((**A**), 5-point scale 0–4) and pruritus ((**B**), 4-point scale 0–3) with IQR (shaded areas) at 0, 3, 6, 12, and 24 h postoperatively. Statistical comparisons were performed using the Mann–Whitney U test (two-sided); raw *p*-values are shown. * *p* < 0.05 (raw *p* before multiplicity correction).

PONV and pruritus scores were low at all assessed time points in both groups (Table 4; Figure 3).

At 6 and 12 h, pruritus scores were nominally lower in the TFP block group compared with the ITM group (raw *p* = 0.025 and *p* = 0.011, respectively); however, these differences did not remain significant after Holm–Bonferroni correction. No consistent between-group differences were observed for PONV at any time point. Although median pruritus scores were 0 in both groups, slight differences in ordinal score distributions were observed because a small number of patients in the ITM group exhibited non-zero pruritus scores at these time points.

The incidence of any PONV or pruritus event was similar between groups (PONV: 20.0% vs. 6.7%, *p* = 0.249; pruritus: 43.3% vs. 33.3%, *p* = 0.596). Full time-point analyses and multiplicity-adjusted results are available in the Supplementary Materials (Tables S3 and S4; Figure S4).

### 3.5. Secondary Outcome: ObsQoR-11

**Figure 4:**
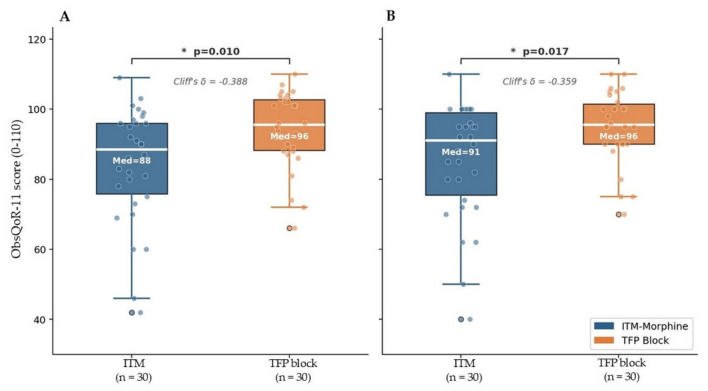
ObsQoR-11. Box-and-whisker plots with individual data points showing ObsQoR-11 scores (0–110; higher = better recovery) at 24 h (**A**) and 48 h (**B**) postoperatively. Boxes represent IQR; horizontal white lines indicate medians; whiskers extend to 1.5× IQR; circles denote outliers; * *p* < 0.05. Statistical comparison by Mann–Whitney U test; Cliff’s delta (δ) reported as effect size (|δ|: < 0.15 negligible; 0.15–0.33 small; 0.33–0.47 medium; > 0.47 large).

Higher ObsQoR-11 scores were observed in the TFP block group compared with the ITM group at both 24 h (median 96 [IQR 88–103] vs. 88 [IQR 76–96]; *p* = 0.010; Cliff’s δ = −0.39) and 48 h (median 96 [IQR 90–102] vs. 91 [IQR 76–99]; *p* = 0.017; Cliff’s δ = −0.36) (Table 5; Figure 4).

Although these differences reached nominal statistical significance, they did not remain significant after Holm–Bonferroni correction for multiple comparisons and should therefore be interpreted as exploratory. Hodges–Lehmann estimates of the median difference were +8.0 points (bootstrap 95% CI +2 to +15) at 24 h, suggesting a potential exploratory between-group difference in postoperative recovery [8], and +7.0 points (95% CI 0 to +15) at 48 h. Effect sizes were within the medium range (0.33 ≤ |δ| < 0.47). Additional exploratory distributional analyses and distributional characteristics are provided in the Supplementary Materials (Table S5; Figure S5).

## 4. Discussion

In this prospective comparative study, bilateral ultrasound-guided TFP block was associated with postoperative analgesic outcomes broadly similar to those observed with ITM with respect to 24 h rescue analgesic requirement following elective caesarean delivery. Within the constraints of the study design and sample size, these findings were consistent with non-inferiority of TFP block within the prespecified analytical framework. In parallel, TFP block was associated with modest improvements in patient-centered recovery, as reflected by higher ObsQoR-11 scores at both 24 and 48 h; however, these differences did not remain statistically significant after adjustment for multiple comparisons and should therefore be interpreted cautiously.

To our knowledge, comparative evaluations of TFP block and ITM incorporating both analgesic and recovery-centered endpoints in the obstetric setting remain limited. Available randomized evidence in related clinical contexts suggests that fascial plane blocks may contribute to opioid-sparing multimodal analgesia strategies [18]. Within this context, the present findings should be regarded as hypothesis-generating and supportive of further prospective evaluation rather than providing definitive evidence of comparative effectiveness.

The findings observed for TFP block within the applied non-inferiority analytical framework warrant careful interpretation. Notably, median 24 h tramadol consumption was zero in both groups, suggesting that the majority of patients achieved adequate analgesia without the need for rescue opioids under the applied multimodal regimen. The rescue rate in the TFP group (16.7%) was numerically lower than in the ITM group (23.3%), and the upper bound of the 95% confidence interval for the risk difference (+13.5%) remained within the pre-specified non-inferiority margin of 15 percentage points, consistent with the prespecified non-inferiority analytical framework. These findings are aligned with the growing body of evidence suggesting that well-placed fascial plane blocks may provide analgesia after caesarean section broadly comparable to outcomes reported with neuraxial opioid-based strategies when integrated into a structured multimodal analgesic regimen [15,19,20].

The ObsQoR-11 difference at 24 h (Hodges–Lehmann estimate +8.0 points) may suggest an exploratory difference in postoperative recovery; however, these findings should be interpreted cautiously given the exploratory nature of the analyses and the absence of a formally established MCID threshold for ObsQoR-11.

Although the confidence interval includes values close to zero and should therefore be interpreted with caution, a greater proportion of patients in the TFP group had higher ObsQoR-11 scores compared with the ITM group at 24 h. Recovery quality in the postpartum period extends beyond analgesia alone, encompassing not only pain and mobility but also the capacity for early neonatal care, breastfeeding, and functional maternal recovery, dimensions that are directly captured by the ObsQoR-11.

The more favorable recovery profile observed in the TFP group may reflect, at least in part, reduced exposure to neuraxial opioid-related adverse effects, particularly pruritus, which—despite not reaching statistical significance after multiplicity correction—was consistently more frequent in the ITM group during the early postoperative period.

The pattern of pruritus observed in this study is broadly consistent with current mechanistic understanding of neuraxial opioid-related pruritus. Neuraxial morphine-induced pruritus is thought to reflect central opioid receptor-mediated mechanisms—effects that are largely independent of histamine signaling and therefore often poorly responsive to antihistamine therapy [6,7]. In the present study, any-event pruritus affected 43.3% of ITM patients versus 33.3% of TFP patients. Although this difference did not reach statistical significance, its direction and magnitude are broadly consistent with published incidence estimates for neuraxial morphine [6,7]. The absence of statistical significance may reflect the modest sample size and the ordinal distribution of pruritus scores rather than excluding a potentially meaningful between-group difference. This interpretation is also in keeping with contemporary data emphasizing the adverse-effect profile of ITM in obstetric populations [18].

Exploratory repeated-measures analysis suggested a consistent overall group effect across time for both resting and movement-evoked pain scores, with no evidence of a group × time interaction. However, absolute NRS differences were small and both groups remained below the rescue threshold throughout the observation period.

These findings should, however, be interpreted in the context of multiplicity adjustment, which attenuated statistical significance across secondary outcomes.

Several limitations of the present study should be acknowledged. First, the sample size of 30 patients per group, although aligned with the pre-specified non-inferiority analytical framework, limits the precision of secondary outcome estimates and contributes to relatively wide confidence intervals. Although a non-inferiority analytical framework was applied, the observational nonrandomized design does not permit formal conclusions regarding non-inferiority or equivalence. In addition, despite generally similar baseline characteristics—including prior caesarean delivery history—residual confounding related to unmeasured factors influencing postoperative pain perception cannot be excluded. Second, the study was conducted at a single center, which may limit generalizability. Third, blinding of the anesthesiologist performing the block was not feasible; although patient and outcome assessor blinding were maintained, this may have introduced a risk of performance or detection bias, particularly for patient-reported outcomes. Fourth, the TFP block technique and local anesthetic dosing regimen used in this study may not be universally standardized, and outcomes may differ with alternative approaches or injectate volumes. Finally, only short-term outcomes (up to 48 h) were assessed, and the durability of the observed effects beyond this period remains uncertain. Accordingly, the present findings should be interpreted cautiously and considered exploratory and hypothesis-generating.

Future studies should incorporate larger sample sizes, multicenter designs, and extended follow-up, including outcomes such as breastfeeding initiation and maternal satisfaction. Dose-optimization studies for TFP block in the obstetric population, as well as direct comparisons with other fascial plane techniques (e.g., quadratus lumborum and erector spinae plane blocks), are also warranted.

## 5. Conclusions

Ultrasound-guided bilateral TFP block was associated with findings consistent with non-inferiority within the prespecified analytical framework relative to ITM for 24 h post-caesarean rescue analgesic requirements, while ObsQoR-11 differences should be interpreted as exploratory. The observed opioid-sparing profile and lower pruritus burden may warrant further evaluation of TFP block within multimodal analgesic strategies for elective caesarean delivery. Given the observational design, sample size, and exploratory nature of secondary outcomes, these findings should be interpreted cautiously and confirmed in larger, multicenter studies.

## Figures and Tables

**Table 1:** Baseline Demographic and Clinical Characteristics.

Variable	ITM (*n* = 30)	TFP Block (*n* = 30)	*p*-Value
Age (years), mean ± SD	29.6 ± 5.3	29.8 ± 5.0	0.970
Weight (kg), mean ± SD	77.7 ± 13.3	80.8 ± 13.5	0.399
Height (cm), mean ± SD	160.3 ± 5.3	162.2 ± 6.5	0.209
BMI (kg/m^2^), mean ± SD	30.2 ± 4.8	30.7 ± 4.9	0.790
Prior caesarean deliveries, mean ± SD	1.9 ± 0.7	1.8 ± 0.6	0.568

Data are mean ± SD. Group comparisons were performed using the Mann–Whitney U test (two-sided), as appropriate. BMI = body mass index; SD = standard deviation.

**Table 2:** Primary Outcome: 24-Hour Rescue Analgesic Requirement and Tramadol Consumption.

Outcome	ITM (*n* = 30)	TFP Block (*n* = 30)	*p*	Effect Size
24 h tramadol, mg—median [IQR]	0 [0–0]	0 [0–0]	0.567 *	—
Rescue analgesic required—*n* (%)	7 (23.3%)	5 (16.7%)	0.748 †	OR 0.66 (95% CI 0.18–2.36)
Tramadol dose (rescue only)—median [IQR]	200 [100–200] mg	200 [100–200] mg	0.718 ‡	—

* Mann–Whitney U test; † Fisher’s exact test; ‡ Mann–Whitney U test among patients requiring rescue analgesia. OR = odds ratio; CI = confidence interval; IQR = interquartile range.

**Table 3:** NRS Pain Scores at Rest and on Movement.

Time	ITM Rest Median [IQR]	TFP Rest Median [IQR]	ITM Movement Median [IQR]	TFP Movement Median [IQR]	*p* (Rest)	*p* (Movement)
0 h	0 [0–0]	0 [0–0]	0 [0–0]	0 [0–0]	0.334	0.334
3 h	2 [1–3]	1 [0–3]	3 [2–3]	2 [0–3]	0.197	0.125
6 h	2 [1–3]	2 [1–3]	3 [2–4]	3 [2–4]	0.383	0.318
12 h	2 [1–3]	1 [0–2]	3 [2–4]	2 [1–3]	0.075	0.156
24 h	1 [1–2]	0 [0–1]	2 [1–3]	2 [1–2]	0.022 *	0.551

Data are presented as median [IQR]. Comparisons were performed using the Mann–Whitney U test (two-sided). * Raw *p* < 0.05; not significant after Holm–Bonferroni correction. IQR = interquartile range.

**Table 4:** PONV and Pruritus Scores Over Time.

Time	ITM PONV	TFP PONV	ITM Pruritus	TFP Pruritus	*p* (PONV)	*p* (Pruritus)
0 h	0 [0–0]	0 [0–0]	0 [0–0]	0 [0–0]	0.161	0.082
3 h	0 [0–0]	0 [0–0]	0 [0–1]	0 [0–0]	0.238	0.063
6 h	0 [0–0]	0 [0–0]	0 [0–0]	0 [0–0]	0.082	0.025 *
12 h	0 [0–0]	0 [0–0]	0 [0–0]	0 [0–0]	0.161	0.011 *
24 h	0 [0–0]	0 [0–0]	0 [0–0]	0 [0–0]	1.000	0.334
Any event	6 (20.0%)	2 (6.7%)	13 (43.3%)	10 (33.3%)	0.249 †	0.596 †

Data are presented as median [IQR] unless otherwise stated. * Raw *p* <0.05; not significant after Holm–Bonferroni correction. † Fisher’s exact test for any-event comparisons. PONV scored 0–4; pruritus scored 0–3.

**Table 5:** ObsQoR-11 at 24 and 48 h postoperatively. Data are presented as median [IQR]. Between-group comparisons were performed using the Mann–Whitney U test; effect size is reported as Cliff’s δ with 95% CI.

Time Point	ITM Median [IQR]	TFP Block Median [IQR]	*p*	Cliff’s δ (95% CI)
ObsQoR-11 at 24 h	88 [76–96]	96 [88–103]	0.010 *	−0.39 (−0.58 to −0.15)
ObsQoR-11 at 48 h	91 [76–99]	96 [90–102]	0.017 *	−0.36 (−0.55 to −0.13)

Data are median [IQR]. Comparisons by Mann–Whitney U test (two-sided). * *p* < 0.05. Cliff’s δ: |δ| < 0.15 negligible; 0.15–0.33 small; 0.33–0.47 medium; >0.47 large. ObsQoR-11 range 0–110 (higher = better). CI = confidence interval.

## Data Availability

The data presented in this study are available in this article and in the Supplementary Materials. Further inquiries can be directed to the corresponding author.

## Supplementary Materials

The following supporting information can be downloaded at: https://www.mdpi.com/article/10.3390/jcm15114157/s1, Table S1: Unadjusted and Adjusted Logistic Regression Models for Rescue Analgesic Requirement; Table S2: Non-Inferiority Analysis for 24-Hour Rescue Analgesic Requirement; Table S3: Holm–Bonferroni-Corrected p Values for Secondary Outcomes; Table S4: Summary of Repeated-Measures Analyses Across Postoperative Time Points; Table S5: Exploratory Distributional Analysis of ObsQoR-11 at 24 and 48 Hours; Figure S1: Summary of Effect Sizes Across All Outcomes; Figure S2: Logistic Regression Analysis for Rescue Analgesia; Figure S3: Non-Inferiority Analysis; Figure S4: Repeated-Measures Analysis of Postoperative Outcomes; Figure S5: Distribution of ObsQoR-11 Scores.

## Abbreviations

The following abbreviations are used in this manuscript:

ASA	American Society of Anesthesiologists
CI	confidence interval
ITM	intrathecal morphine
IQR	interquartile range
NRS	Numerical Rating Scale
ObsQoR-11	Obstetric Quality of Recovery-11
OR	odds ratio
PONV	postoperative nausea and vomiting
TFP	transversalis fascia plane

## References

[1] Carvalho B., Butwick A.J. (2017). Postcesarean delivery analgesia. Best Pract. Res. Clin. Anaesthesiol..

[2] Sultan P., Gutierrez M.C., Carvalho B. (2011). Neuraxial morphine and respiratory depression: Finding the right balance. Drugs.

[3] Dahl J.B., Jeppesen I.S., Jørgensen H., Wetterslev J., Møiniche S. (1999). Intraoperative and postoperative analgesic efficacy and adverse effects of intrathecal opioids in patients undergoing cesarean section with spinal anesthesia: A qualitative and quantitative systematic review of randomized controlled trials. Anesthesiology.

[4] Bollag L., Lim G., Sultan P., Habib A.S., Landau R., Zakowski M., Tiouririne M., Bhamidipati S., Carvalho B. (2021). Society for Obstetric Anesthesia and Perinatology: Consensus Statement and Recommendations for Enhanced Recovery After Cesarean. Anesth. Analg..

[5] Crowe G., Atterton B., Roofthooft E., Joshi G.P., Rawal N., Wu C., Sauter A.R., Bonnet M.P., Lucas D.N., Van de Velde M. (2026). Pain management after elective caesarean section under neuraxial anaesthesia: An updated systematic review and procedure-specific postoperative pain management (PROSPECT) recommendations. Anaesthesia.

[6] Ganesh A., Maxwell L.G. (2007). Pathophysiology and management of opioid-induced pruritus. Drugs.

[7] Szarvas S., Harmon D., Murphy D. (2003). Neuraxial opioid-induced pruritus: A review. J. Clin. Anesth..

[8] Ciechanowicz S., Setty T., Robson E., Sathasivam C., Chazapis M., Dick J., Carvalho B., Sultan P. (2019). Development and evaluation of an obstetric quality-of-recovery score (ObsQoR-11) after elective Caesarean delivery. Br. J. Anaesth..

[9] Wang P., Chen X., Chang Y., Wang Y., Cui H. (2021). Analgesic efficacy of ultrasound-guided transversus abdominis plane block after cesarean delivery: A systematic review and meta-analysis. J. Obstet. Gynaecol. Res..

[10] Tulgar S., Serifsoy T.E. (2018). Transversalis fascia plane block provides effective postoperative analgesia for cesarean section: New indication for known block. J. Clin. Anesth..

[11] Aydin M.E., Bedir Z., Yayik A.M., Celik E.C., Ates I., Ahiskalioglu E.O., Ahiskalioglu A. (2020). Subarachnoid block and ultrasound-guided transversalis fascia plane block for caesarean section: A randomised, double-blind, placebo-controlled trial. Eur. J. Anaesthesiol..

[12] Elsharkawy H., El-Boghdadly K., Barrington M. (2019). Quadratus Lumborum Block: Anatomical Concepts, Mechanisms, and Techniques. Anesthesiology.

[13] Tan H.S., Taylor C., Weikel D., Barton K., Habib A.S. (2020). Quadratus lumborum block for postoperative analgesia after cesarean delivery: A systematic review with meta-analysis and trial-sequential analysis. J. Clin. Anesth..

[14] Sharawi N., Carvalho B., Habib A.S., Blake L., Mhyre J.M., Sultan P. (2018). A systematic review evaluating neuraxial morphine and diamorphine-associated respiratory depression after cesarean delivery. Anesth. Analg..

[15] Hussain N., Brull R., Thaete L., Fuller S., D’Souza R.S., Mankinen-Abdallah Y., Essandoh M.K., Weaver T.E., Abdallah F.W. (2025). The analgesic effects of novel fascial plane blocks compared with intrathecal morphine after Caesarean delivery: A systematic review and meta-analysis. Br. J. Anaesth..

[16] Cadirci D.Y., Aydin M.E., Ahiskalioglu E.O., Çelik E.C., Yayik A.M., Çoruh Ş., Şenocak G.N.C., Ciftci B., Ahiskalioglu A. (2025). Intrathecal morphine versus transversalis fascia and transversus abdominis plane blocks combination on obstetric quality of recovery after cesarean delivery: A randomized controlled trial. BMC Anesthesiol..

[17] von Elm E., Altman D.G., Egger M., Pocock S.J., Gøtzsche P.C., Vandenbroucke J.P., STROBE Initiative (2007). The Strengthening the Reporting of Observational Studies in Epidemiology (STROBE) statement: Guidelines for reporting observational studies. PLoS Med..

[18] Lustig A., Carvalho B., Haim S., Guo N., Greenberger C., Karol D., Aptekman B., Weiniger C.F. (2026). Prospective study of postoperative respiratory depression metrics after cesarean delivery among women receiving intrathecal morphine. Anesth. Analg..

[19] Baghirzada L., Walker A., Yu H.C., Endersby R. (2024). The analgesic effect of transversalis fascia plane block after caesarean section under spinal anaesthesia with intrathecal morphine: A randomised controlled trial. Anaesthesia.

[20] Dost B., De Cassai A., Bugada D., Balzani E., Karapinar Y.E., Beldagli M., Ozkal Yalin M.S., Tulgar S., Ahiskalioglu A. (2025). The analgesic effect of transversalis fascia plane block after cesarean delivery: A systematic review and meta-analysis with trial sequential analysis. Minerva Anestesiol..

