# Supplementary material for: Ultrasound-Guided Transversalis Fascia Plane Block Versus Intrathecal Morphine for Post-Cesarean Analgesia: A Prospective Observational Comparative Cohort Study Incorporating a Non-Inferiority Analytical Framework
Source: J Clin Med. 2026 May 28;15(11):4157. doi: 10.3390/jcm15114157 (PMC13258360; doi:10.3390/jcm15114157)
Supplement: Supplementary file 1 [file jcm-15-04157-s001.zip › jcm-4303822-supplementary.pdf]

SUPPLEMENTARY MATERIALS

Ultrasound-Guided Transversalis Fascia Plane Block versus Intrathecal Morphine for Post-Cesarean Analgesia: A Prospective Observational Comparative Cohort Study Incorporating a Non-Inferiority Analytical Framework

Supplementary Methods

Detailed Statistical Analysis

Supplementary analyses were conducted to further characterize the primary and secondary outcomes. For the primary endpoint (24-hour tramadol consumption), a two-part (hurdle) analytical framework was applied:

(i) logistic regression for rescue analgesic requirement (binary outcome)

(ii) regression analysis restricted to patients requiring rescue analgesia.

Adjusted analyses included maternal age, body mass index, and parity as clinically relevant covariates. Repeated-measures analyses for pain scores were performed using appropriate models including group, time, and group × time interaction terms. Multiplicity adjustment across secondary outcomes was performed using the Holm–Bonferroni method.

These supplementary analyses are exploratory in nature and should be interpreted as supportive rather than confirmatory.

Supplementary Tables

Table S1. Unadjusted and Adjusted Logistic Regression Models for Rescue Analgesic Requirement. Unadjusted and adjusted logistic regression models for 24-hour rescue analgesic requirement. Model 1 presents unadjusted estimates; Model 2 includes adjustment for maternal age, body mass index, and parity. Odds ratios (ORs) with 95% confidence intervals (CIs) are reported.

Variable	Beta	SE	OR	95% CI	p value
--- Model 1: Unadjusted ---					
Group: TFP vs ITM	-0.420	0.653	0.657	0.183-2.363	0.520

--- Model 2: Adjusted (Group + Age + BMI + Parity) ---					
Group: TFP vs ITM (adj)	-0.329	0.670	0.719	0.193-2.675	0.623
Age (per SD)	-0.305	0.385	0.737	0.347-1.566	0.427
BMI (per SD)	0.017	0.346	1.018	0.517-2.003	0.960
Parity (per SD)	0.567	0.367	1.762	0.859-3.617	0.122

OR = odds ratio; CI = 95% confidence interval. Estimates are presented for unadjusted and adjusted models.

Table S2. Non-Inferiority Analysis for 24-Hour Rescue Analgesic Requirement. Results of non-inferiority analysis comparing TFP block and ITM for 24-hour rescue analgesic requirement. Estimates are presented relative to the pre-specified non-inferiority margin.

Parameter	ITM	TFP block	Estimate	95% CI / Result
Rescue rate (n/N)	7/30 (23.3%)	5/30 (16.7%)	--	--
Risk Difference (TFP-ITM)	--	--	-6.7 pp	-26.8% to +13.5%
Risk Ratio	--	--	0.71	0.25-2.00
Odds Ratio	--	--	0.66	0.18-2.36
NI margin (delta-0)	--	--	15.0%	Pre-specified
Upper 95% CI bound	--	--	+13.5%	< 15% NI margin
NI test z / one-sided p	--	--	z = 2.105	p = 0.018
Interpretation within the analytical framework	--	--	Consistent with non-inferiority under the prespecified analytical framework	Upper CI < delta-0

pp = percentage points. Results are presented relative to the pre-specified non-inferiority margin. The upper bound of the 95% confidence interval is shown in relation to the margin.

Table S3. Holm–Bonferroni-Corrected p Values for Secondary Outcomes. This table presents raw and Holm–Bonferroni–corrected p values for all secondary outcomes, including NRS pain scores (rest and movement), PONV, pruritus, and ObsQoR-11 at all predefined time points.

Outcome	Time	Raw p	Holm-corrected p
NRS rest	0 h	0.334	1.000
NRS rest	3 h	0.197	1.000
NRS rest	6 h	0.383	1.000
NRS rest	12 h	0.075	1.000
NRS rest	24 h	0.022*	0.423
NRS movement	0 h	0.334	1.000
NRS movement	3 h	0.125	1.000
NRS movement	6 h	0.318	1.000
NRS movement	12 h	0.156	1.000
NRS movement	24 h	0.551	1.000
PONV	0 h	0.161	1.000
PONV	3 h	0.238	1.000
PONV	6 h	0.082	1.000
PONV	12 h	0.161	1.000
PONV	24 h	1.000	1.000
Pruritus	0 h	0.082	1.000
Pruritus	3 h	0.063	1.000
Pruritus	6 h	0.025*	0.444
Pruritus	12 h	0.011*	0.231
Pruritus	24 h	0.334	1.000
ObsQoR-11	24 h	0.010*	0.221
ObsQoR-11	48 h	0.017*	0.333

* Raw $p < 0.05$. NRS = Numerical Rating Scale; PONV = postoperative nausea and vomiting; ObsQoR-11 = Obstetric Quality of Recovery-11

Table S4. Summary of Repeated-Measures Analyses Across Postoperative Time Points. Summary of repeated-measures analysis across postoperative time points (0, 3, 6, 12, 24 h). Mann–Whitney U statistics were converted to z-scores and combined using Stouffer's method to assess overall group effects. Group × time interaction was evaluated using Cochran's Q test.

Outcome	Overall group z	Overall p	Interaction Q	df	Interaction p	Interpretation
NRS at Rest	2.790	0.005*	2.281	4	0.684	Group effect
NRS on Movement	2.093	0.036*	1.169	4	0.883	Group effect
PONV Score	0.992	0.321	0.295	4	0.990	No group effect
Pruritus Score	1.422	0.155	4.661	4	0.324	No group effect

* $p < 0.05$. Mann–Whitney U statistics were converted to z-scores and combined using Stouffer's method. Group × time interaction was evaluated using Cochran's Q test.

Table S5. Exploratory Distributional Analysis of ObsQoR-11 at 24 and 48 Hours. Hodges–Lehmann estimation and bootstrap CI were used to explore and contextualize the magnitude of between-group differences.

Parameter	ITM	TFP block	Difference (TFP–ITM)	Clinical Interpretation
ObsQoR-11 at 24 h, median [IQR]	88.5 [76–96]	95.5 [88–103]	+7.0 pts	Exploratory difference
Hodges–Lehmann estimate	--	--	+8.0 pts	Exploratory finding
Bootstrap 95% CI (24 h)	--	--	+2 to +15	CI excludes zero
ObsQoR-11 at 48 h, median [IQR]	91.0 [76–99]	95.5 [90–102]	+4.5 pts	Modest difference
Hodges–Lehmann estimate	--	--	+7.0 pts	Exploratory trend
Bootstrap 95% CI (48 h)	--	--	0 to +15	CI includes zero

Higher recovery score proportion above ITM median (24 h)	23.3%	43.3%	+20.0 pp	Potential recovery trend

At 24 h, the Hodges–Lehmann estimate (+8.0 points; 95% CI +2 to +15) suggested an exploratory between-group difference with a confidence interval excluding zero. At 48 h, the estimate (+7.0 points; 95% CI 0 to +15) suggested a more modest exploratory trend.

Supplementary Summary of Key Analyses

Analysis	Key finding	Direction	Clinical interpretation	Manuscript use
GEE – NRS rest	z = 2.79, p = 0.005	Difference between groups	Difference across time points	Results – secondary
GEE – NRS movement	z = 2.09, p = 0.036	Difference between groups	Difference across time points	Results – secondary
Adjusted logistic regression	adj-OR 0.72, p = 0.623	Not significant	No evidence of confounding	Methods support
Non-inferiority analysis	Upper 95% CI +13.5%	Within NI margin	Within prespecified NI margin	Primary outcome
ObsQoR-11 exploratory analysis (24 h)	HL +8.0 (CI +2 to +15)	Exploratory difference	CI excludes zero	Primary outcome
ObsQoR-11 exploratory analysis (48 h)	HL +7.0 (CI 0 to +15)	Exploratory trend	CI includes zero	Secondary

Supplementary Figures

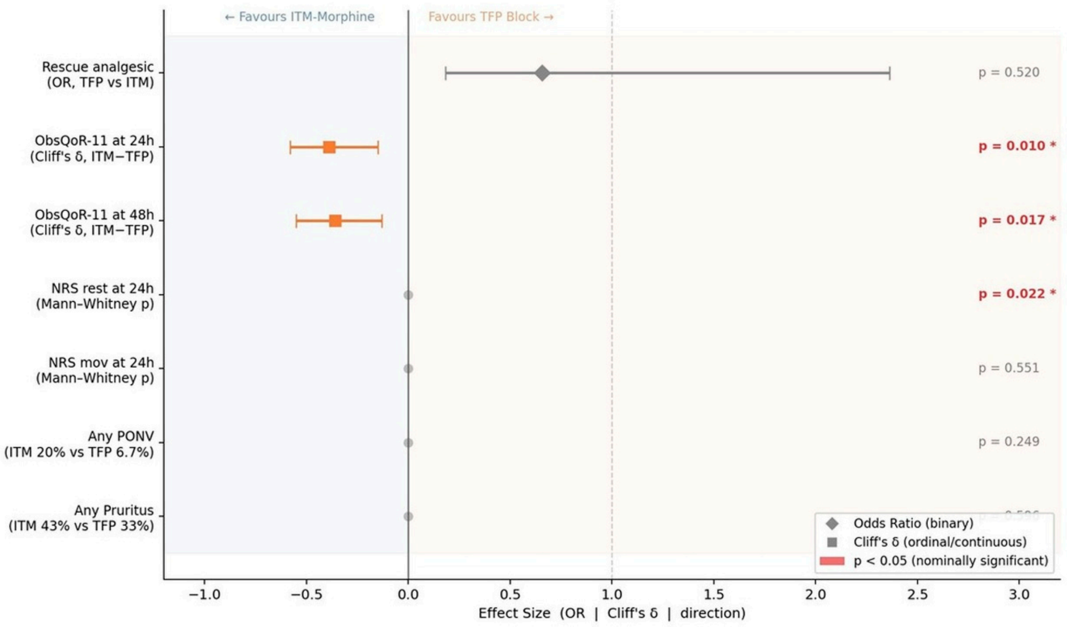

Figure S1. Summary of Effect Sizes Across All Outcomes. Forest-style summary of effect sizes for primary and secondary outcomes. Odds ratios (OR) with 95% confidence intervals are shown for rescue analgesic requirement, and Cliff's delta (δ) for ObsQoR-11 outcomes. For ordinal outcomes (NRS, PONV, pruritus), Mann–Whitney U test p values are displayed. Effect sizes are presented with their corresponding confidence intervals where applicable.

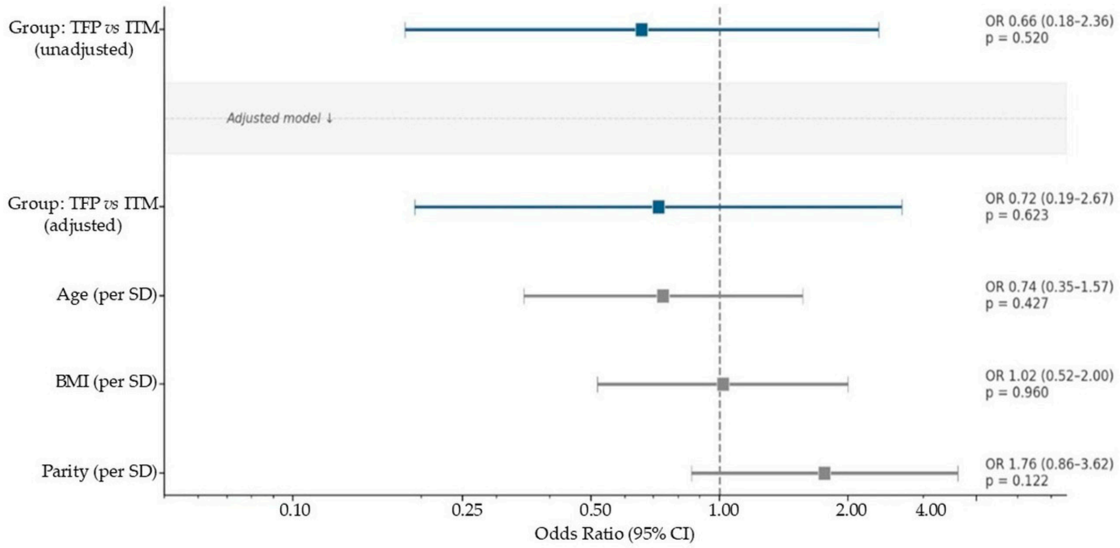

Figure S2. Logistic Regression Analysis for Rescue Analgesia. Forest plot of odds ratios (log scale) for 24-hour rescue analgesic requirement based on unadjusted and adjusted logistic regression models. Estimates are presented with 95% confidence intervals.

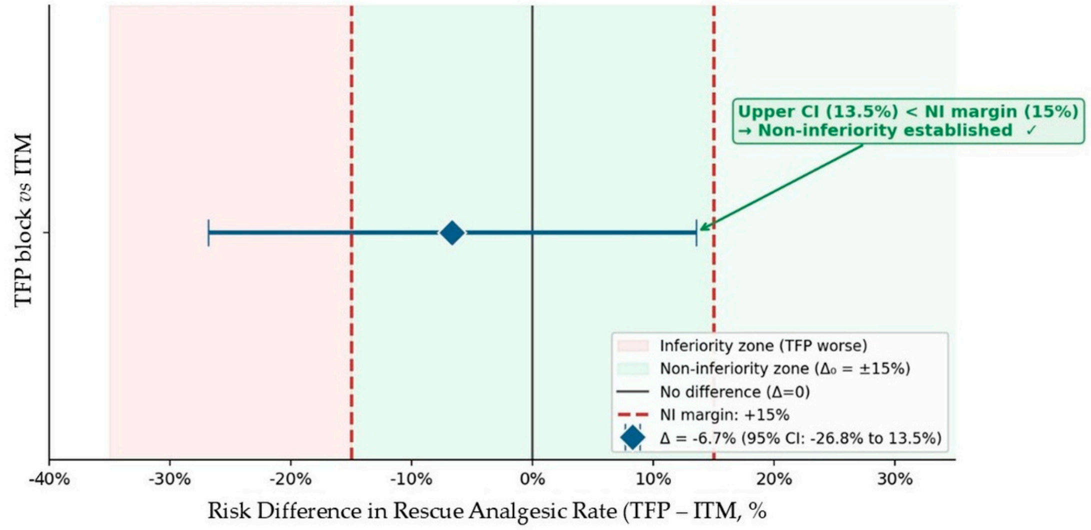

Figure S3. Non-Inferiority Analysis. Non-inferiority plot for 24-hour rescue analgesic requirement. The risk difference (TFP – ITM) is presented with 95% confidence intervals relative to the pre-specified non-inferiority margin. Interpretation is presented within an exploratory observational non-inferiority analytical framework and should not be interpreted as formal confirmatory evidence.

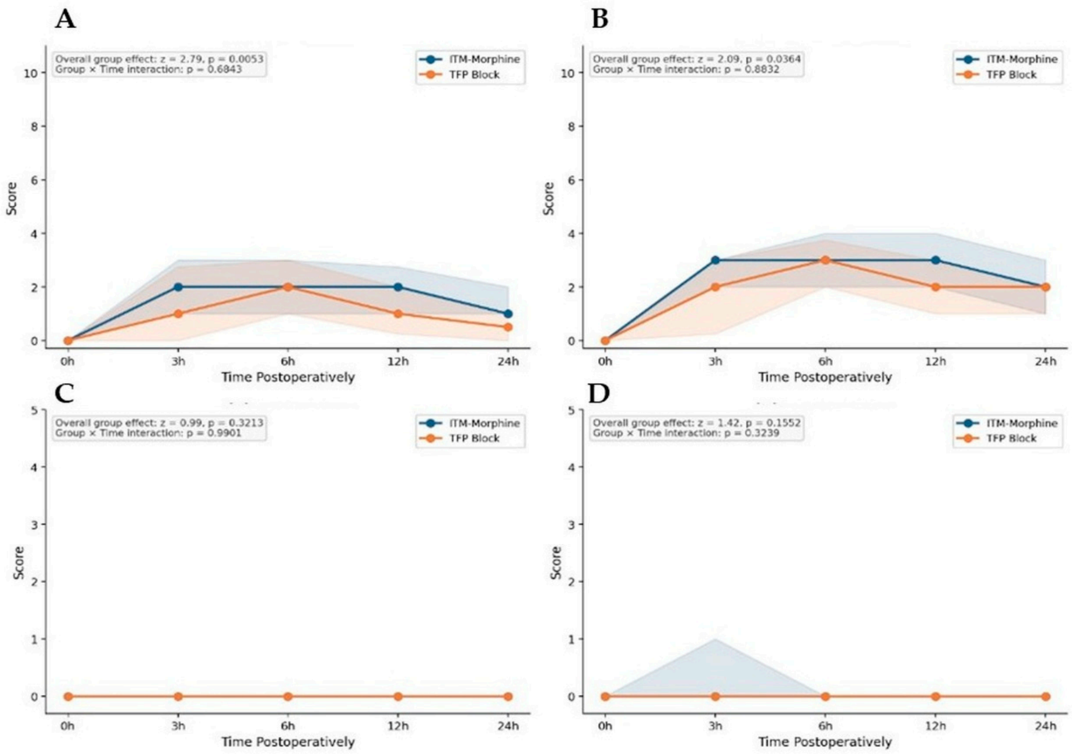

Figure S4. Repeated-Measures Analysis of Postoperative Outcomes. (A) NRS pain scores at rest; (B) NRS pain scores on movement; (C) postoperative nausea and vomiting (PONV) scores; and (D) pruritus scores across postoperative time points (0, 3, 6, 12, and 24 h). Data are presented as medians with interquartile ranges. Insets display overall group effects (Stouffer z and p values) and group × time interactions (Cochran's Q and p values).

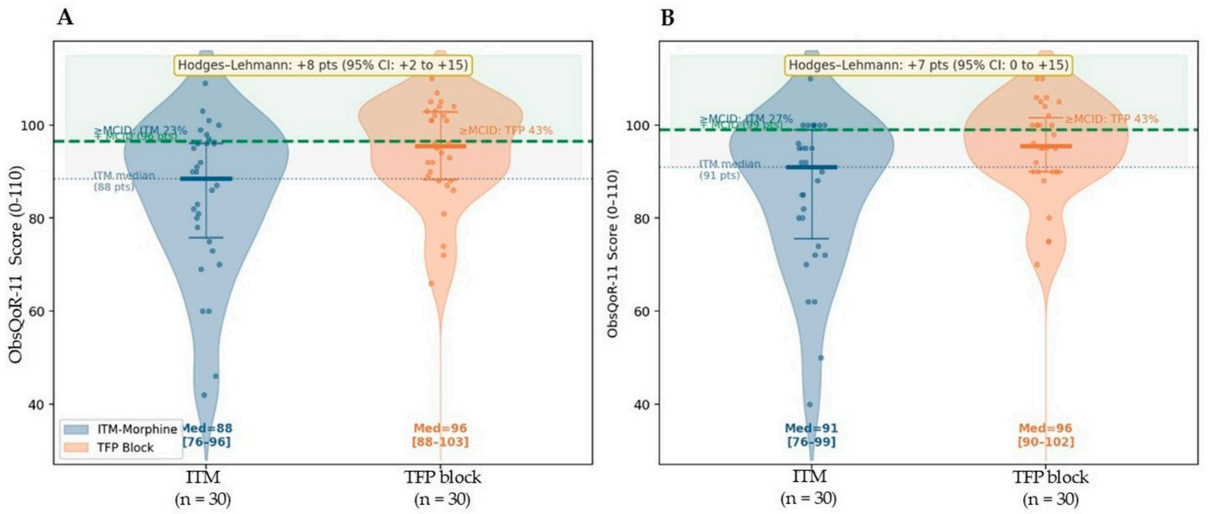

Figure S5. Distribution of ObsQoR-11 Scores. (A) ObsQoR-11 scores at 24 hours; (B) ObsQoR-11 scores at 48 hours. Violin plots display score distributions with individual data points, medians, and interquartile ranges. Hodges–Lehmann estimates with 95% confidence intervals are shown. The dashed horizontal line represents the exploratory reference threshold. Labels displayed within the figure were retained from the original exploratory visualization and do not represent formally validated MCID thresholds.
